# To what extent do site-based training, mentoring, and operational research improve district health system management and leadership in low- and middle-income countries: a systematic review protocol

**DOI:** 10.1186/s13643-016-0239-z

**Published:** 2016-04-27

**Authors:** Zakaria Belrhiti, Andrew Booth, Bruno Marchal, Roosmarijn Verstraeten

**Affiliations:** Department of Management and Economy, National School of Public Health, Rabat, Morocco; School of Health & Related Research (ScHARR), University of Sheffield, Sheffield, UK; Health Services Organisation Unit, Institute of Tropical Medicine, Antwerp, Belgium; Nutrition and Child Health Unit, Institute of Tropical Medicine, Antwerp, Belgium

**Keywords:** Site-based training, Mentoring, Operational research, Best fit framework synthesis, District health, Management, Leadership, Low- and middle-income countries

## Abstract

**Background:**

District health managers play a key role in the effectiveness of decentralized health systems in low- and middle-income countries. Inadequate management and leadership skills often hamper their ability to improve quality of care and effectiveness of health service delivery. Nevertheless, significant investments have been made in capacity-building programmes based on site-based training, mentoring, and operational research. This systematic review aims to review the effectiveness of site-based training, mentoring, and operational research (or action research) on the improvement of district health system management and leadership. Our secondary objectives are to assess whether variations in composition or intensity of the intervention influence its effectiveness and to identify enabling and constraining contexts and underlying mechanisms.

**Methods:**

We will search the following databases: MEDLINE, PsycInfo, Cochrane Library, CRD database (DARE), Cochrane Effective Practice and Organisation of Care (EPOC) group, ISI Web of Science, Health Evidence.org, PDQ-Evidence, ERIC, EMBASE, and TRIP. Complementary search will be performed (hand-searching journals and citation and reference tracking).

Studies that meet the following PICO (Population, Intervention, Comparison, Outcome) criteria will be included: P: professionals working at district health management level; I: site-based training with or without mentoring, or operational research; C: normal institutional arrangements; and O: district health management functions. We will include cluster randomized controlled trials, controlled before-and-after studies, interrupted time series analysis, quasi-experimental designs, and cohort and longitudinal studies. Qualitative research will be included to contextualize findings and identify barriers and facilitators.

Primary outcomes that will be reported are district health management and leadership functions. We will assess risk of bias with the Cochrane Collaboration’s tools for randomized controlled trials (RCT) and non RCT studies and Critical Appraisal Skills Programme checklists for qualitative studies. We will assess strength of recommendations with the GRADE tool for quantitative studies, and the CERQual approach for qualitative studies. Synthesis of quantitative studies will be performed through meta-analysis when appropriate. Best fit framework synthesis will be used to synthesize qualitative studies.

**Discussion:**

This protocol paper describes a systematic review assessing the effectiveness of site-based training (with or without mentoring programmes or operational research) on the improvement of district health system management and leadership.

**Systematic review registration:**

PROSPERO CRD42015032351

**Electronic supplementary material:**

The online version of this article (doi:10.1186/s13643-016-0239-z) contains supplementary material, which is available to authorized users.

## Background

### Description of the condition

Decentralization has been a common healthcare reform in low- and middle-income countries (LMICs) since the 1950s and the early 1960s [1]. By decentralization we mean the transfer of authority or delegation of power in public planning, management, and decision-making from the national level to sub-national levels [1–3].

A common form of decentralization in LMICs is deconcentration, which has been defined as the handing over of some administrative authority from the central level of government to the district level of, for instance, a ministry of health. Deconcentration in the health sector aims at establishing a local district management team with clearly defined administrative duties and a degree of discretion that would enable local officials to manage without constant reference to ministry headquarters within a limited administrative area (e.g. health district) [1, 4].

However, inadequate leadership and management capacities of district health managers often hamper their ability to improve the quality, effectiveness, and efficiency of health service delivery, which in turn may contribute to a decreased use of healthcare services by the local population [5, 6].

### Description of the intervention

There has been a significant investment in capacity-building programmes aiming at developing and maintaining essential competencies required for optimal public health and effective health service delivery [2, 6]. These capacity-building programmes are activities and processes that improve the ability of staff within organizations to carry out stated objectives [7].

**Fig. 1:**
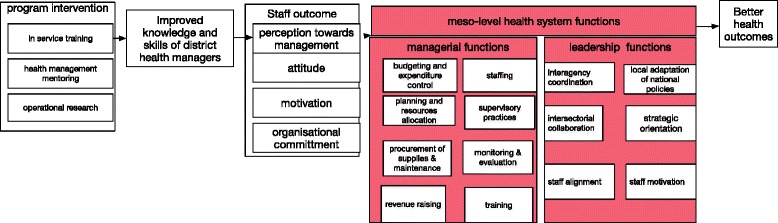
Logic model of site-based training, mentoring, and operational research intervention in a district health system

In many LMICs, site-based training^1^ and mentoring programmes have been implemented in order to strengthen competencies and provide supportive mentorship for local district health managers (see Fig. 1).

Mentoring (M) is a flexible learning and teaching process that serves specific objectives of a health programme. Health management mentoring involves spending time with managers in their local environment to assist them in their day-to-day challenges. Mentorship is defined as the dynamic, reciprocal relationship in a work environment between an advanced career incumbent (mentor) and a beginner (mentee) [8]. It aims at promoting the development of both the mentor and mentee. Mentoring is recognized as a catalyst for facilitating career selection, advancement, and productivity [8, 9].

Site-based training (SBT) (also called in-service training) involves training current district health managers in their work settings. Such training may enable health managers to run health districts in an integrated manner through sound management of primary and secondary health services, team building, and supervision [10]. SBT has been, and will remain, a significant investment in developing and maintaining essential competencies required for optimal public health in local district level service settings [11].

Operational research (OR) is “the search for knowledge on interventions, strategies, or tools that can enhance the quality, effectiveness, or coverage of programmes in which the research is being done” [12].

Action research (AR) is “a method used for improving practice. It involves action, evaluation, and critical reflection and – based on the evidence gathered – changes in practice are then implemented.” [13].

Both operational research and action research in this review are understood as a tool for capacity-building and as a close collaborative investigation between researchers and district health managers (DHM). In this paper, we will refer to both strategies as operational research.

Complex interventions are combinations of interacting and interdependent actions. The overall effect of the intervention could be higher or lower when interactions are synergistic or antagonistic, respectively. Our review will illuminate the effectiveness of site-based training intervention alone (single intervention) versus site-based training interventions associated with mentoring and/or operational research activities (multicomponent intervention): i.e. (SBT+M+OR) or (SBT+OR) or (SBT+M).

This review will identify the relationship between the intensity of the intervention (number of components) and the effect size. It will seek to reconcile conflicting evidence of the effectiveness of single intervention versus multi-component intervention [14, 15].

### How the intervention might work

The World Health Organization (WHO) has defined four factors that enable the improvement of district health systems management: (1) there should be an adequate number of trained managers; (2) managers should have appropriate competencies; (3) there should be support systems to provide managers with the resources they need to carry out their responsibilities, including systems for planning and budgeting as well as human resource management; and (4) the environment in which the managers function should enable them to carry out their responsibilities.

Site-based training will improve the DHM competencies while mentoring DHM will provide the necessary supportive environment to help them carry out their day-to-day responsibilities (factors 2 and 4 of the WHO framework) [6]. Meanwhile, operational research fosters the ability of DHM to make sense of national policies, to translate them into operational terms, and to integrate them into their day-to-day practices [16]. Operational research connects researchers with DHM in their work settings. Researchers reflect on their ways of supporting managers, while managers identify work-related issues, analyse them, take action, and reflect on their action [17, 18].

We built a logic model (see Fig. 1) based on previous research to help us focus on our review question and guide data collection processes [19–21]. Our review is focused on:

- P: professionals working at district health management level
- I: site-based training with or without mentoring AND/OR operational research
- C: normal institutional arrangements
- O: district health management functions (see Table 1)

**Table 1:** District management and leadership functions

Primary outcomes *District management functions* refer to the functions of budgeting and expenditure control, supervisory practices, staffing, planning and resource allocation, procurement of supplies, maintenance, local adaptation of national policies, and revenue raising and training. *Leadership functions* stand for interagency coordination, inter-sectorial collaboration, strategic orientation, and staff alignment and motivation Managerial and leadership functions are what Gilson (2012) refers to as the meso-level health system functions.	

### Why it is important to do this review

A recent systematic review [11] has shown no or low-quality evidence of site-based training on the improvement of knowledge, competencies, and health outcomes. However, specific district management and leadership functions were not covered by this review.

This review will inform policymakers and educational institutions involved in site-based training and mentoring about the appropriate educational methods to use, constraints to avoid, and key mechanisms that enhance the effectiveness of these interventions and contextual factors that improve the performance of district health management.

### Objectives

Our objective is to evaluate the available evidence on the effectiveness of site-based training, mentoring, and operational research on the improvement of district health system management and leadership (see Table 1). We will specifically evaluate site-based training intervention alone (single intervention) versus site-based training interventions associated with mentoring and/or operational research activities (multicomponent intervention).

We acknowledge that this intervention is multifaceted [14, 15] and complex in nature, as it is implemented in social systems characterized by human agency, uncertainty, and unpredictability. Hence, our secondary objectives are to identify the enabling or constraining contexts, mechanisms for the effectiveness of this intervention. We will report broader generalizable trends across multiple settings and will devise a “best fit framework” that will help policymakers understand how and why the intervention works.

We acknowledge that we may not review other relevant outcomes due to practical considerations within a limited timeframe (see Additional file 1).

### Criteria for considering studies for this review

#### Population

##### Inclusion criteria

The population to be included in this review concerns DHM. We define DHM as health officers actually involved in district health management and spending some of their time in management and/or administrative functions within the health district. These include district medical officers, nursing officers, health inspectors, administrators, counsellors from the district health committee, representatives of a hospital (hospital directors), district administrators, representatives of clinics, medical assistants, or local government promotion officers. Also included are health workers who carry out administrative tasks besides their clinical practice, such as medical doctor, nurse practitioners,^2^ clinical officers^3^, or primary healthcare officers.

##### Exclusion criteria

Medical and nursing students will be excluded from the review. Community health workers^4^ are also excluded because they are not involved in the management of health districts.

### Intervention(s)

#### Intensity

##### Inclusion criteria

The intervention is a complex multifaceted intervention (i.e. combination of different strategies) that is composed of site-based training, with or without mentoring, and/or operational research at district level. Site-based training is the principal component, and mentoring and operational research are optional components of the intervention. However, we would consider studies where site-based training is accompanied by mentoring or operational research programmes.

##### Exclusion criteria

Traditional in-class training, pre-service training, or medical education will be excluded. Training and mentoring focusing on vertical programmes will also be excluded because of our focus on health district systemic functioning.

#### Who delivers the intervention

Independent researchers, academics, or local managers might deliver the intervention.

### Comparators

Control groups concern districts that are not the site of site-based training, mentoring, and operational research interventions, delivering regular care.

### Outcomes

#### Primary outcomes

Intermediate outcomes, as depicted in the logic model (box in red in Fig. 1), are the major focus of this review. We have identified major outcomes that are crucial for an effective functioning of local health systems. These outcomes are categorized into district health management and leadership functions. We validated these outcomes with content experts^5^ along with end users of this review.^6^ These outcomes are grouped into two categories, district health management and leadership functions (see Table 1).

#### Secondary outcomes

This review intends to address the following secondary outcomes:

- Mechanisms underlying the effect of the intervention
- Enabling and constraining contextual factors
- Comparison of effectiveness of single intervention versus multicomponent intervention

### Types of study

In order to assess the effectiveness of site-based training, mentoring, and operational research interventions on the performance of district health system managers, we will rely on study designs that are capable of demonstrating a causal relationship namely the following: cluster randomized controlled trials, controlled before-and-after studies(CBA), interrupted time series(ITS), quasi-experimental design, cohort studies, and longitudinal studies.

We will also collect and extract information from qualitative studies linked to, or associated with, included studies. Qualitative research and economic evaluation studies will be used in the review to help contextualize findings and identify barriers and facilitators. Qualitative research will help to identify how the intervention might work (underlying mechanisms), within a particular local historical and institutional context.

Reviews and meta-analyses will be excluded, but eligible studies identified from within existing reviews will be included.

### Context

#### Inclusion criteria

Only interventions that are delivered in district health systems in LMICs will be included in this review.

#### Exclusion criteria

We will exclude studies carried out in high-income countries (HICs) because their health district models are far different from the district health system functioning and staff involved. Therefore, evidence from HICs will be hardly transferrable to LMICs. Also, studies that have not been conducted within decentralized health district levels will be excluded.

### Search strategy

Our search strategy relies on three elements (population, intervention, and context) from the PICOC (Population, Intervention, Comparison, Outcome, Context) framework [22]. We adopted a sensitive search strategy rather than a specific one to scan a whole range of studies in the specific field of health systems research.

Since we also will be gathering qualitative evidence, the search strategy has to be inclusive in order not to miss relevant papers. Therefore, we used a combination of thesaurus terms and free text terms (Shaw, Booth et al. 2004) (Fretheim, Oxman et al. 2009—see Appendix 1). Search limits and sources to be searched are depicted in Tables 2 and 3, respectively.

**Table 2:** Search limits

Study designs	((((((((((((((“evaluation”[All Fields]) OR realist[All Fields]) OR “Program Evaluation”[Mesh]) OR “Evaluation Studies” [Publication Type]) OR “cohort studies”[MeSH Terms]) OR (“Outcome and Process Assessment (Health Care)”[Mesh])) OR Evalu*) OR interview) OR Qualitative) OR “Qualitative Research” [Mesh]) OR Randomized Controlled Trial [Publication Type]) OR Random*) OR Randomly [tiab]) OR Randomized controlled trial [pt]) OR Interrupted Time Series Analysis
Publication types	Grey literature will not be reviewed
Date of publication	From 1978^a^
Language	English, French, Arabic and Spanish
Other limits	Filter humans

^a^Reviewers and content experts will review all the articles that have been published after the Alma Ata declaration (1978) fostering the role of primary healthcare

**Table 3:** Sources to be searched

Databases	(1) MEDLINE, (2) PsycInfo, (3) Cochrane Library, (4) CRD database (DARE), (5) Cochrane Effective Practice and Organisation of Care (EPOC) group, (6) ISI Web of Knowledge, (7) Health Evidence.org, (8) PDQ-Evidence, (9) ERIC, (10) EMBASE, (11) TRIP
Grey literature	We will review only published articles.
Other sources	Reference tracking, citation tracking, hand-searching journals

## Data collection and analysis

### Study selection

A team of two reviewers^7^^,^^8^ will be involved in selecting studies. We are using Endnote as reference manager software. Title and abstract screening will be operationalized using Microsoft Excel sheets to record the process, including justifying reasons for exclusion. Full-text articles will be obtained in cases of doubt. In cases of disagreement between the reviewers, we will consult a third reviewer.

A kappa coefficient will be measured to make sure that discordance does not impact on the validity of the selection process. The review team includes two experts in evidence-informed decision-making.^9^ Since we are interested in implementation gaps, qualitative studies and process evaluations will be obtained and considered alongside the included studies. We mean by process evaluation research that is used to assess fidelity, quality, and reach of implementation; to clarify causal mechanisms; and to identify contextual factors associated with variation in outcomes [23]. Data from multiple reports of the same study will be collated.

### Data extraction

Two reviewers will carry out data extraction with external validation by mentors^10^ of the review. Additional searches for process evaluations and qualitative and implementation studies will be performed using citation searching to inform an understanding of the context, mechanisms, barriers, facilitators, cost, and sustainability of the intervention implementation. Authors will be contacted if implementation data are not included in the published articles or in case of missing data. Relevant data will be collected for each type of intervention (site-based training, mentoring, operational research) according to the data extraction form presented below.

### Data extraction form

1. Author names, journal, year
2. Study design
3. Unit of analysis
4. Sampling methods
5. Type of intervention:
  - Organizational intervention
  - Professional (educational intervention)
6. Participants (profession, administrative position, level of training, clinical specialty, age, time since graduation)
7. Setting (location; country; district level, primary or secondary level; rural of urban area)
8. Intervention characteristics

For each type of intervention (mentoring, site-based training, and action research), data will be collected according to the data depicted in Table 4.

**Table 4:** Intervention characteristics

Country Year Duration of the programme, Frequency Programme components Underlying theory of change Barriers Facilitators Educational methods Intervention model Participants Programme Goal Programme evaluation Evaluation results Outcomes Sustainability Length of post intervention follow-up	

We will report the frequency and time during which outcome measurement was done. Length of post intervention follow-up will also be collected. Duplicate or multiple reports of the same study will be assembled and compared for duplication, completeness, and possible contradictions. Data will be extracted using a Microsoft Excel spreadsheet form. EndNote and RevMan software will be used for data storage and analysis. Authors will be contacted by mail in case of missing data. Measures of effects in included studies that will be reported are relative risk (RR) for dichotomous variables and categorical measures such as Likert scales and means or changes of means over time for continuous data.

### Proposed quantitative data synthesis

We will use RevMan software to perform meta-analysis for quantitative data if similar outcomes and measurement scales were used. A test of heterogeneity (*I*^2^ statistic) will be carried out in order to test the relevance of meta-analysis and to inform decisions about the choice of either fixed effects or random effects models of meta-analysis. We will also explore whether effect size differs between single intervention (SBT) and multicomponent intervention (SBT and Mentoring AND/OR operational research). Meta regression, using Revman 5, could be used to examine conditional relationship among predictors (intensity of the intervention and other subgroup variables) and effect size magnitude.

### Proposed qualitative data synthesis

We will first use best fit framework (BFF) synthesis to synthetize qualitative evidence gathered from included studies. This methodology fits the analysis of organizational policies and procedures. It is also appropriate for research within a limited time frame, with specific questions, and with heterogeneous outcomes [24, 25]. The main purpose of the BFF is to describe and interpret what is happening in specific contexts. It goes beyond identifying insights from individual case studies to reporting broader generalizable trends across multiple settings. Therefore, BFF helps practitioners design suitable action plans that address system-wide issues.

**Fig. 2:**
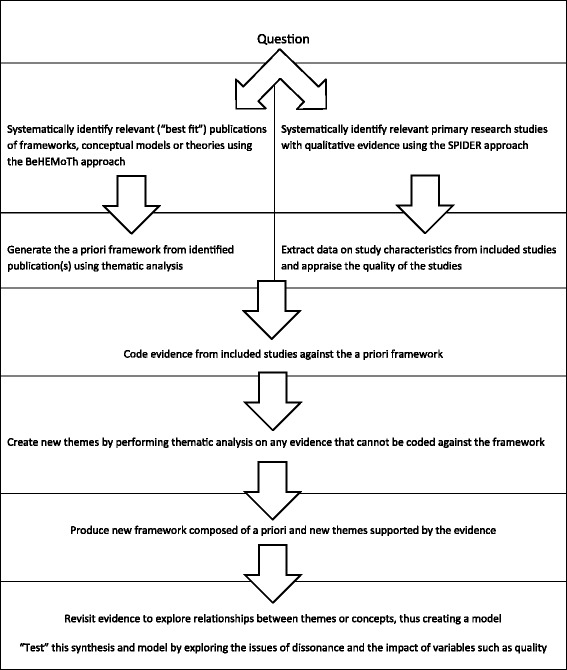
Best fit framework synthesis steps [25]

BFF is about choosing a conceptual framework that suits the question of the review, using it as the basis for an initial coding framework. In response to the evidence gathered, the framework is subsequently altered, so that the final model is a modified framework that includes both modified factors, for example those achieving additional granularity, and new factors not anticipated by the a priori framework. The revised framework thus becomes a more generalizable framework [25, 26]. The steps of BFF are depicted in Fig. 2 [25]. We will use the logic model, depicted in Fig. 1, as our a priori framework similarly to an increasing number of systematic reviews [27–29]. Logic models help to highlight key contextual elements and to focus on the underlying programme theory. We do not claim that it is an ideal model, only that it fits the purpose of this review.

We will identify relevant framework publications using truncated terms (theor^11^*, concept*,^12^ framework*^13^ or model*^14^) in our reference management database of included studies. Supplementary search will be carried out on external databases using BeHEMoTh (Behaviour of interest, Health context, Exclusions, Models or Theories) template (see search strategy in Appendix 2) [25]. Study data will be extracted against the concepts and subcategories of the framework.

### Quality assessment strategy

For RCTs and non RCTs studies (CBA, ITS) the Cochrane Collaboration tool [30] and EPOC Tools [31] will be used, respectively, for assessing risk of bias. Critical Appraisal Skills Programme (CASP) checklist tools will be used for qualitative studies [32]^15^. We will assess the quality of studies and categorize them as low risk of bias, unclear risk of bias, and high risk of bias. These categories will be assigned respectively if there is an unlikely plausible risk of bias that could alter confidence in the results, plausible bias that raise a doubt of the validity of the results, or plausible bias that seriously weakens the confidence in results.

Besides, overall strength of recommendations will be assessed using the Grades of Recommendation, Assessment, Development and Evaluation (GRADE) approach. Quality of body of evidence will be categorized into four categories (high, moderate, low, and very low) based on the degree of likelihood of specific risk of bias, publication bias indirectness (surrogate outcomes, indirect comparison, difference in population, difference in intervention), inconsistency, and imprecision.

We will identify risk of bias using specific criteria [33]; estimate publication bias using funnel plots of study results [34]; and assess likelihood of inconsistency with criteria such as variation of point estimate, absence of minimal overlap of confidence interval, statistical test for heterogeneity, and *I*^2^ [35, 36].

For qualitative studies, the CASP tool will allow us to question the validity of the results, the quality of the analysis process, and the relevance for local context. To ensure consistency with the GRADE approach, we will use the corresponding CERQual approach for the qualitative studies. This will allow us to categorize the evidence into the same four categories (high, moderate, low, and very low) based on the likely confidence in the findings (methodological limitations, publication bias, relevance, coherence, and adequacy of data) [37].

### Reporting

This protocol was reported according to the Preferred Reporting Items for Systematic Reviews and Meta-analyses (PRISMA)-P Statement for reporting systematic review protocols (see Additional file 2) [38].

## Appendix 1

### MEDLINE (PubMed) search strategy

We used the search terms MeSH and free terms intervention + population + LMIC^18^ context+ decentralized local health system context

(((((((((“Hands on Training” OR training method* OR In service Training OR “In service training” OR Training OR “Continuing professional education” OR “On the job training”)) OR (“Education, Continuing”[Mesh] OR “education” [Subheading] OR “Staff Development”[Mesh] OR “Inservice Training”[Mesh]))) OR (((Coaching OR supportive supervision OR Mentee OR mentor*)) OR mentors [MeSH Terms])) OR (((Participatory action research OR operational research OR learning by doing OR Research Healthcare OR Healthcare, Research OR Health Services OR Action Research OR Research, Action OR Health Services Research/organization & administration*)) OR (“Health Services Research”[Mesh] OR “Empirical Research”[Mesh]))) AND ((((((administrative OR Management) AND (personnel OR employee* OR team OR position* OR system* OR support OR Staff OR Position)) OR District health managers OR district medical officer OR nursing officer OR health inspector OR administrator OR district health committee OR hospital directors OR district administrator OR representative of clinic medical assistant OR local government promotion officer OR primary health care officers OR Nurse practitioner OR Clinical officer)) OR Institutional management team[MeSH Terms]) OR Administrative personnel[MeSH Terms])) AND Developing Countries[MeSH Terms]) OR ((((((((Afghanistan* OR afghan OR Gambia* OR Niger* OR Benin* OR guine* OR Rwanda* OR burkina Faso* OR guinea Bissau* OR bissau OR sierra Leone* OR Burundi* OR haiti* OR Somalia* OR Cambodia* OR Korea* OR south Sudan* OR Sudan* OR central African* republic OR Liberia* OR Tanzania* OR chad* OR madagascar* OR Togo* OR comoros* OR malawi* OR Uganda* OR congo, dem. rep OR zaire OR mali OR zimbabwe OR eritrea* OR mozambi* OR ethiopi* OR nepal OR armenia OR indonesia OR samoa OR bangladesh OR kenya OR sao tome and principe OR sao tome and principe OR golf of guinea OR bhutan OR kiribati OR senegal OR bolivia OR kosovo OR solomon islands OR cabo verde OR kyrgyz republic OR sri lanka OR cameroon OR lao pdr OR sudan OR congo, rep. OR lesotho OR swaziland OR cote d’ivoire OR mauritania OR syrian arab republic OR syria OR Djibouti* OR Micronesia*, Fed. Sts. OR Micronesia* OR tajikistan OR Egypt*, arab rep. OR Egypt* OR moldova OR timor-leste OR Timor* OR El Salvador* OR salvadoran OR Morocc* OR Ukrain* OR Georgia* OR myanmar OR burma OR burmese OR uzbekistan OR Uzbek* OR Ghana* OR Nicaragua* OR Vanuatu* OR Guatemala* OR nigeria* OR Vietnam* OR Guyan* OR Pakistan* OR west bank and gaza OR Palestin* OR Hondura* OR papua new Guinea* OR yemen, rep. OR Yemen* OR India* OR Philippine* OR Zambia* OR Albania* OR Fiji* OR Namibia* OR Algeria* OR Gabon* OR Palau* OR american Samoa* OR Grenada* OR panama * OR Angola* OR iran, islamic rep OR IRAN* OR Paraguay* OR Azerbaijan* OR Iraq* OR Peru* OR belarus* OR Jamaica* OR Romania* OR Belize* OR Jordan* OR Serbia* OR bosnian OR bosnia and herzegovina OR Kazakhstan* OR south Africa* OR Botswana* OR Leban* OR St. Lucia* OR brazil* OR Libya* OR st. vincent and the grenadines OR Grenadian* OR Bulgaria* OR Macedonia* FYR OR Suriname* OR China* OR chinese* OR malaysia* OR thailand* OR Colombia* OR Maldiv* OR tonga* OR costa Rica* OR marshall islands OR tunisia* OR Cuba* OR mauritius OR Mauritian* OR turkey OR turkish OR Dominica* OR dominican, rep OR mexico OR Mexican* OR Turkmenistan* OR dominican republic OR Dominican* OR Mongolia* OR tuvalu OR Ecuador* OR Montenegr*)))))) AND (Decentralis* OR Decentraliz* OR Local OR local level OR District) AND (management” OR health system* OR team* OR health team* OR Health service* OR health system OR level management’ OR level health system*) AND ((“1978/01/01”[PDat] : “3000/12/31”[PDat]) AND Humans[Mesh] AND (Arabic[lang] OR English[lang] OR French[lang] OR Spanish[lang])))

## Appendix 2

### Search strategy for relevant frameworks (BeHEMoTh)

(((((((CAPACITY BUILDING) OR health system strengthening) OR strengthening health system) AND ((health district AND (leadership OR management)))) NOT ((“surveillance model” OR “disease model*” OR “measurement model”OR “Care model”)) AND ((Theor* OR Model* OR Concept* OR Framework*))))))

**Table:**

Behaviour of interest	health district AND (leadership OR management)
Health context	CAPACITY BUILDING) OR health system strengthening) OR strengthening health system)
Exclusion	“surveillance model” OR “disease model*” OR “measurement model” OR “Care model
Models of theories	Theor* OR Model* OR Concept* OR Framework*)))))

## Additional files

Additional file 1: Systematic review time frame. (PDF 169 kb) Additional file 2: PRISMA-P (Preferred Reporting Items for Systematic review and Meta-Analysis Protocols) 2015 checklist: recommended items to address in a systematic review protocol*. (PDF 147 kb)

## Abbreviations

AR: action research
BeHEMoTh: Behaviour of interest, Health context, Exclusions, Models or Theories
BFF: best fit framework
CBA: Control before and after studies
CASP: Critical Appraisal Skills Programme
DARE: Database of Abstracts of Reviews of Effects
DHM: district health managers
EPOC: Cochrane Effective Practice and Organisation of Care
ERIC: Education Resources Information Center
EVIDENT: Evidence Informed Decision-Making in Health and Nutrition
HICs: high-income countries
ITS: Interrupted Time Series
LMICs: low- and middle-income countries
PDQ-Evidence: “Pretty Darn Quick” evidence
OR: operational research
PICO: Population, Intervention, Comparison, Outcome
PICOC: Population, Intervention, Comparison, Outcome, Context
RCT: randomized controlled trials
SBT: site-based training
WHO: World Health Organization

## Footnotes

1 Site-based training is also called on-the-job training, hands-on training, and continuing education, which means training that takes place in workplace settings.

2 Nurse practitioners: nurses who are specially trained to assume an expanded role in providing medical care under the supervision of a physician

3 Clinical officer (formerly called medical assistant) is a medical separate healthcare provider who has clinical duties (diagnosis, treatment of patients in primary healthcare, assessment, management, and transfer of surgical patients) and administrative duties at respective health centres.

4 Community members who connect healthcare beneficiaries with providers in order to promote access to health

5 Prof. Bart Criel, Dr. Geneviève Michaux, and Prof. Bruno Marchal from the Institute of Tropical Medicine, Antwerp

6 Members of the RIPSEC consortium. RIPSEC is an EU-funded programme that aims at implementing a site-based training intervention, mentoring, and operational research in four health districts in the Democratic Republic of Congo.

7 Zakaria Belrhiti, National School of Public Health, PhD student at Institute of Tropical Medicine

8 Issam Bennis, National School of Public Health, PhD student at Institute of Tropical Medicine

9 Andrew Booth, University of Sheffield, and Roos Verstraeten, Institute of Tropical Medicine, Antwerp

10 Ibid

11 Theory, theories, theoretical

12 Concept, concepts, and conceptual

13 Framework, frameworks

14 Model, models

15 Critical Appraisal Skills Programme (CASP). Qualitative Research Checklist. 2013 available at http://www.casp-uk.net/#!checklists/cb36
