# Supplementary material for: To what extent do site-based training, mentoring, and operational research improve district health system management and leadership in low- and middle-income countries: a systematic review protocol
Source: Syst Rev. 2016 Apr 27;5:70. doi: 10.1186/s13643-016-0239-z (PMC4847191; doi:10.1186/s13643-016-0239-z)
Supplement: Additional file 1: — Systematic review time frame. (PDF 169 kb) [file 13643_2016_239_MOESM1_ESM.pdf]

Additional file 1: Systematic review time frame

**Task**	**Completion date (D/M/Y)**
Focus question	01/9 /2015
Draft protocol	01/10/2015
Scoping search	15/11/2015
Final protocol	15/12/2015
Registering in PROSPERO	1/1/2016
Full searches	15/02/2016
Study selection	1/03/2016
Order papers	1/04/2016
Quality assessment	30 /4/2016
Data extraction	30/5/2016
Data synthesis	30/6/2016
Draft review submission	30/7/2016
Final review submission	30/8/2016
